# Changes in bud bank and their correlation with plant community composition in degraded alpine meadows

**DOI:** 10.3389/fpls.2023.1259340

**Published:** 2023-10-13

**Authors:** Yuan Li, Gensheng Bao, Peng Zhang, Xiaoyun Feng, Jingjuan Ma, Hainian Lu, Hongxiao Shi, Xiaoxing Wei, Bingming Tang, Kai Liu

**Affiliations:** ^1^ Academy of Animal and Veterinary Medicine, Qinghai University, Xining, China; ^2^ State Key Laboratory of Sanjiangyuan Ecology and Plateau Agriculture and Animal Husbandry, Qinghai University, Xining, China; ^3^ Grassland Research Institute of the Chinese Academy of Agricultural Science, Hohhot, China; ^4^ Qinghai Provincial Grassland Station, Xining, China

**Keywords:** alpine meadow, degraded grassland, bud bank, vegetative reproduction, diversity index

## Abstract

Bud banks are considered a crucial factor in regulating the species composition of grassland communities and maintaining the ecological function of alpine grasslands. However, few studies have paid attention to the dynamic changes of bud banks from undisturbed to severely degraded alpine meadows. Therefore, this study examined the correlations between plant diversity and bud bank traits at different stages of alpine meadows degradation. Grass biomasses and plant diversity were found to be highest in moderately degraded meadows, and sedge biomasses were highest in lightly degraded meadows. Lack of disturbance and moderate disturbance by herbivores increased the bud bank density of alpine meadows. Consistent with the changes in bud bank density, bud bank diversity was highest in undisturbed meadows. The structural equation model indicated that the densities of rhizome and the densities and diversities of tiller buds play crucial roles in facilitating the greater diversity of the plant community. Our findings suggest that the diversities and densities of rhizome and tiller buds in the degradation stages are synchronized with changes in plant diversity, and in the regenerative ability of bud banks, which largely determine the outcome of restoration in degraded meadows. These findings could provide a frame of reference for effectively restoring degraded alpine regions by regenerating bud banks. The potential driving force and renewal capacity of bud banks should be taken into account in restoring the Qinghai-Tibet Plateau’s degraded meadow.

## Introduction

Bud banks are defined as collections of buds, such as rhizome, tiller and corm buds, that have the potential to carry out vegetative reproduction (Qian et al., 2014; Liu et al., 2022). Rhizome buds mainly generate from the belowground horizontal rhizomes of dicotyledons and monocotyledons; tiller buds originate from the bases of plant tiller nodes; and corm buds develop from the tips of underground stems (Klimešová and Klimeš, 2007). Previous studies have shown that bud banks play a fundamental role in regulating vegetation communities and plant diversity - especially for plant communities with low seed yields, because seed germination has a negligible contribution to plant regeneration (Clarke et al., 2013; Dong et al., 2015). For example, an alpine meadow with a dense sod layer was dominated by perennial clonal plants. Based on this observation, it can be argued that changes in plant composition, population regeneration, and community dynamics mainly rely on plant bud banks rather than on soil seed banks (Vítová et al., 2017; Wu et al., 2020a).

The reproductive and regenerative capacities of plant bud banks in alpine regions are affected by variable environmental conditions, such as severe drought, fire and disturbances by herbivores (e.g. trampling and feeding) (Enright and Mille, 2007; Klimešová and Klimeš, 2007; Willand et al., 2013; VanderWeide and Hartnett, 2015). Carter et al. (2012) found that drought reduces the density of belowground bud and that the bud densities of grasses and forbs differ in sensitivity to water-deficiency conditions. Bud banks are also reduced in number as mean precipitation decreases (Dalgleish and Hartnett, 2006). Furthermore, fire has positive effects on regulating bud bank density (Benson et al., 2004; Dalgleish and Hartnett, 2009; Russell et al., 2019; Alexandre et al., 2022). For example, Russell et al. (2019) found that grass bud renewal activity is stimulated by burning. Similarly, Alexandre et al. (2022) found that seasonal fires play a vital role in bud bank dynamics in the Brazil’s Cerrado savannah and that bud bank densities decrease in the dry season. Some buds can develop into new branches in the rainy season. In tallgrass prairies, the total density of bud banks in burnt grassland is twice that of unburnt grassland (Dalgleish and Hartnett, 2009; Benson et al., 2004). Additionally, herbivores can affect bud banks by removing bud resources or stimulating bud outgrowth (Dalgleish and Hartnett, 2009; Harmony et al., 2009; Ott et al., 2019). For example, Harmony et al. (2009) found that grazing and trampling by herbivores markedly reduces the bud density of heavily grazed tallgrass prairies in North America. Dalgleish and Hartnett (2009) suggested that buds renewal rate increases, but bud density decreases in grazed grassland, compared to un-grazed grassland. Therefore, long-term or intensive grazing by herbivores may result in reducing the reproductive capacities of underground bud banks, which may explain the decrease in or loss of degraded grasslands’ self-recovery capacities (Dalgleish and Hartnett, 2009; Ott et al., 2019). Few studies, however, have paid attention to the dynamics of bud density among the different stages of grassland degradation.

Alpine meadows are the most important grassland component of the Qinghai-Tibet Plateau, playing a crucial role in maintaining biodiversity and ecosystem balance, conserving soil and water and regulating global climate change (Yang et al., 2022). However, alpine meadows demonstrate a tendency toward extensive degradation due to the combined effects of climate change, unreasonable anthropogenic disturbances, overgrazing, and rodent burrowing (Min et al., 2011; Fayiah et al., 2020). Previous studies have indicated that bud banks should be regarded as a driving factor of disturbances to plant self-regeneration in perennial grassland ecosystems, and they can be used as effective indicators of the dynamic succession of plant communities (Wang et al., 2008; Rusch et al., 2011; VanderWeide and Hartnett, 2015). Hartnett et al. (2006) attributed 99% of vegetation renewal to the bud banks of perennial caespitose grasses in North American grasslands. Ott and Hartnett (2012) reported that active buds largely determine grasslands’ potential renewal, and that bud bank structure and dynamics differ among functional groups, including C3 and C4 species. Interspecific differences in bud bank dynamics also drive the dynamic patterns of aboveground plants. Similarly, Eighty-four percent of the Alps’ grasslands are occupied by cloned plants (Wellstein and Kuss, 2011). Furthermore, the density and type of bud banks also demonstrated varied responses to grassland degradation. For instance, total bud banks and the density of rhizome buds correlate positively with total aboveground biomass in lightly degraded (LD) grasslands, while tiller bud density correlates positively with plant diversity in the non-degraded grassland (Yang et al., 2022). However, few studies have clearly elucidated the changes in bud bank type, density and diversity in responding to the structures and functions of grassland communities at different stages of degradation.

The aim of the present study was to examine the dynamic changes in bud bank density and diversity from undistributed meadows to severely degraded (SD) alpine meadows and to clarify the correlation between bud banks and plant diversity at different stages of degradation. Therefore, we conducted a field experiment to investigate the diversity of plant communities and bud banks in degraded alpine meadows. The study addressed the following question: (1) How do plant species diversity and functional group biomass change in differently degraded alpine meadows? (2) How does bud bank density respond to different stages of alpine meadows degradation? (3) Are the effects of alpine meadow degradation on bud bank dynamics consistent with the changes in plant community? The knowledge obtained from this study is helpful in understanding the potential role of bud banks in the restoration of degraded meadows and in predicting the dynamics of plant communities in degraded meadows based on bud banks driving plant regeneration.

## Materials and methods

### Study site

We conducted this study in Youganning, a town of in the Mongolian Autonomous County of Henan, Qinghai Province, China (34°C44′18"N, 101°C36′31"E, 3523 m), which occupies to the eastern edge of the Qinghai-Tibet Plateau. The experimental site, which has an area of approximately 10 ha, has been a site for the long-term monitoring and annual investigation of grassland communities since 2018. The region has a plateau continental monsoon climate and an average annual temperature of 0.8°C, ranging from -14.6°C in January to 7.9°C in July. Its annual sunlight duration ranges from 2551.8 h to 2577.2 h, and its total amount of daylight radiation ranges from 139.75 kcal to 152.48 kcal. Annual precipitation averages 598 mm, and the plant growth season lasts five months, from May to September (Du, 2015). The vegetation is alpine meadow, which is dominated by clonal, perennial herbaceous plants, including *Kobresia humilis*, *Elymus nutans*, *Festuca ovina*, *Poa pratensis*, *Carex tristachya*, and *Kobresia capillifolia*, and perennial weeds composed of companion species such as *Potentilla anserina*, *Ligularia virgaurea*, *Polygonum viviparum*, *Morina chinensis* and *Elsholtzia densa*. Five plots with an area of 0.5 ha were selected, as follows, in accordance with Li et al. (2016) classification standard for degraded grasslands: undisturbed (ND), lightly (LD), moderately (MD), heavily (HD), and black-soil-type (SD) degraded meadows (**Table 1**). The distance between plots was at least 5 km (Lin et al., 2016). To ensure the identical grazing intensity of different stages of degradation in designed experimental plots, each plot was enclosed by fence at the beginning of the experiment.

**Table 1:** Detailed information of sampling sites in different degraded alpine meadows.

Number of experimental sites	Degraded degree	Coverage (%)	Proportion of edible forages (%)	Dominating species	Grazing intensity	Duration of grazing
1	Undisturbed meadow (ND)	>85	>80	*Kobresia humilis*, *Kobresia capillifolia*, *Carex tristachya*	0.00 sheep/ha	No grazing
2	Lightly degraded meadow (LD)	70~85	55~80	*Carex tristachya*, *Festuca ovina*, *Elymus nutans*	1.33 sheep/ha	All-year grazing
3	Moderately degraded meadow (MD)	50~70	30~55	*Potentilla anserina*, *Festuca sinensis*, *Carex tristachya*	4.00 sheep/ha	All-year grazing
4	Heavily degraded meadow (HD)	30~50	10~30	*Elymus nutans*, *Poa pratensis*, *Potentilla anserina*, *Ligularia virgaurea*	6.67 sheep/ha	All-year grazing
5	“Black-soil-type” degraded meadow (SD)	<30	<10	*Potentilla anserina*, *Morina chinensis*, *Elsholtzia densa*	11.25 sheep/ha	All-year grazing

### Investigation of plant community traits with alpine meadows of different degrees of degradation

In mid-August 2021, plant community traits were investigated at each plot during the plant growing season. Five replicate subplots with a size of 0.5 × 0.5 m were randomly selected from each plot to measure the height and coverage of each species in the subplot; simultaneously, the stems and leaves of each species were harvested from the surface of the ground (Zhu et al., 2016). The stems and leaves of each species were oven dried to a constant weight at 80°C and then weighed (Ji et al., 2020).

The plant diversities of the degraded alpine meadows were determined by calculating the Margalef index (*R*_m_), the Simpson index (*D*), the Shannon-Wiener index (*H*) and the Pielou index (*E*), using the following formulae (Du et al., 2021):


$$\text{Plant relative importance value }(P_{i})=(\text{relative height}+\text{relative coverage}+\text{relative biomass})/3$$

$$\text{Margalef}:R_{m}=(S-1)/lnN$$

$$\text{Simpson}:D=1-\sum_{i=1}^{S}P_{i}^{\;2}$$

$$\text{Shannon}-\text{Wiener}:H=-\sum_{i=1}^{S}\left(P_{i}\;ln\;P_{i}\right)$$

$$\text{Pielou}:E=-\sum_{i=1}^{S}(P_{i}\;ln\;P_{i})/ln\;S,$$

where *P_i_* is the relative importance value of the *i* species, *N* is the total number of species in the plot, and *S* is the number of species in each subplot.

### Examination of bud banks in degraded alpine meadows

To investigate bud bank type and density, three samples (20 cm long × 20 cm wide × 30 cm deep) were randomly selected from the top soil layer and associated vegetation plants and manually excavated with a spade from different degraded grassland sites (Qian et al., 2017). Buds with soil were taken to the laboratory and immersed in a bucket with an inner diameter of 25 cm and a height of 40 cm at room temperature for 24 h. To avoid any damage to the buds, the immersed samples were then gently washed with running water and carefully brushed with a banister brush to clear rhizosphere soil. Plant species were identified and recorded on the basis of their morphological features, either spikelets or inflorescence, and the densities of rhizome, tiller, and corm buds were recorded (Dalgleish and Hartnett, 2009; Kleyer et al., 2008). Furthermore, the degraded alpine meadows’ bud diversities were determined by calculating the Margalef, Simpson, Shannon-Wiener, and Pielou indexes using the following formulae (Sun et al., 2020):


$$P_{i}=n_{i}/N$$

$$\text{Margalef}:R_{m}=(S-1)/lnN$$

$$\text{Simpson}:D=1-\sum_{i=1}^{S}P_{i}^{\;2}$$

$$\text{Shannon}-\text{Wiener}:H=-\sum_{i=1}^{S}\left(P_{i}\;ln\;P_{i}\right)$$

$$\text{Pielou}:E=-\sum_{i=1}^{S}(P_{i}\;ln\;P_{i})/ln\;S,$$

where *P_i_* is the proportion of individual species *i* among species, *N* is the total number of individual species in the sample, and S is the number of species in the sample.

### Statistical analysis

One-way analysis of variance (ANOVA) was performed to compare the biomass, plant, and bud diversity indexes among the degraded meadows. The bud number within the area of 0.5 × 0.5 m was transformed into 1 m^2^, and one-way ANOVA was used to determine the difference in bud bank density among the degraded alpine meadows. All data are represented as mean ± standard error.

A redundancy analysis (RDA) was performed to analyze the correlation between plant and bud bank diversities (Chandregowda et al., 2018). Furthermore, a structural equation model (SEM) was used to analyze the effects of the density and diversity of bud banks and plant biomass indicators on the diversity levels of plant communities at different levels of degradation. Prior to the SEM analysis, all indicators were screened by correlation analysis, and indicators with high collinearity were deleted. Biomasses of the plant functional groups, bud bank density, and the diversity index were considered as the key factors. Different degradation stages and the plant diversity of alpine meadows were designated as initial and target variables, respectively (Grace et al., 2010). A piecewise SEM module in the R Programming Language was selected to construct piecewise structural equations. All data were analyzed using the IBM Statistical Product and Service Solution (SPSS) software (version 25.0; SPSS China, Shanghai, China), and all figures were produced using SigmaPlot 14.0.

## Results

### Biomasses and diversity index values of plant communities in the degraded alpine meadows

**Figure 1:**
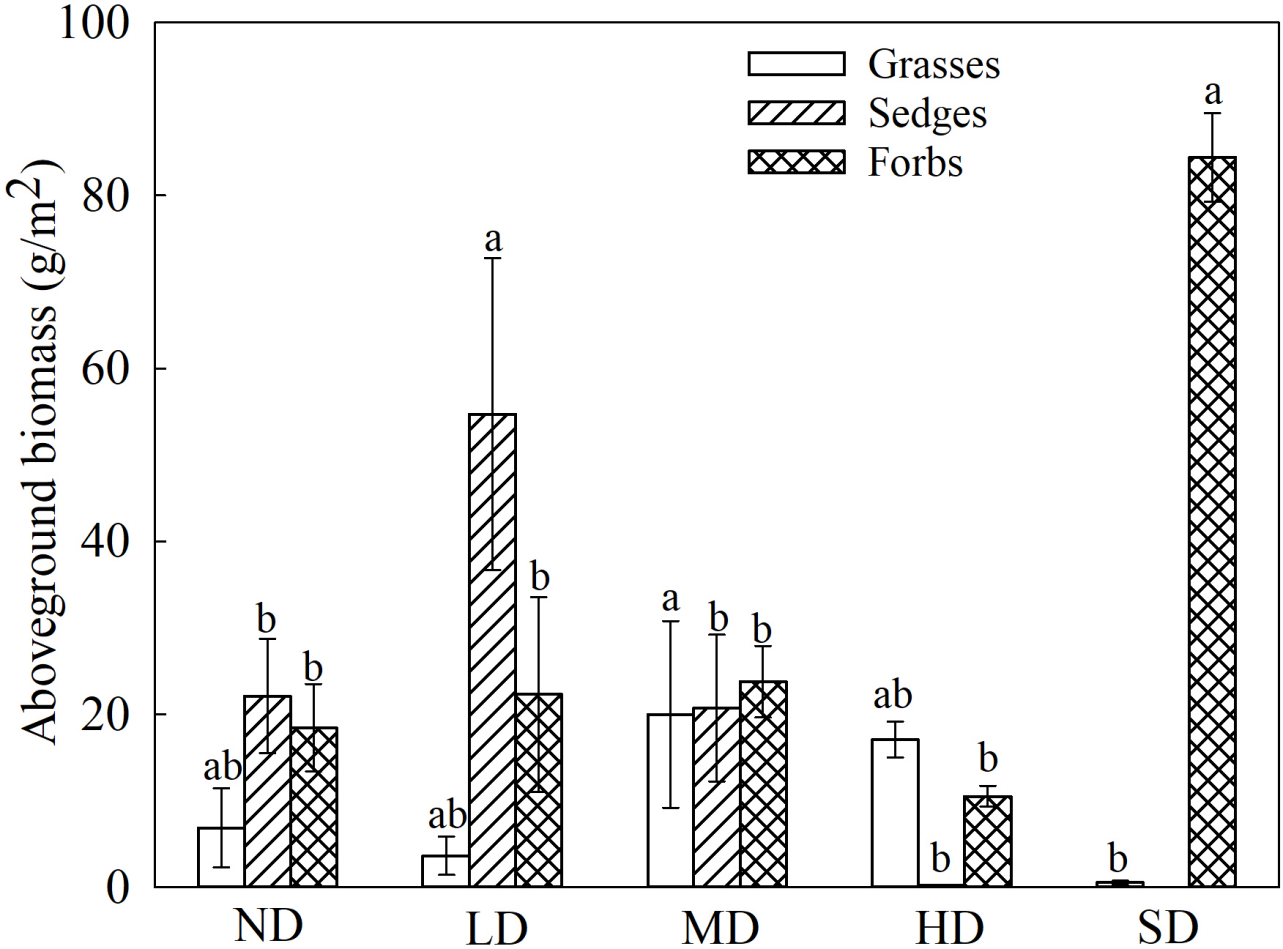
Changes in plant aboveground biomass in degraded alpine meadows. The different lowercase letters indicate that the aboveground biomass of the functional groups has a significant difference at the 0.05 level among the different degradation stages.

Significant differences in biomass and diversity index values were found among the degraded alpine meadows (**Table 2**, **Figure 1**; *P<* 0.05). The Simpson and Shannon-Wiener index values in the MD were higher than those in the LD, HD, and SD meadows (**Table 2**; *P*< 0.05) but did not significantly differ from those in the ND meadows (**Table 2**; *P* > 0.05). Furthermore, the Shannon-Wiener index values in the ND meadows were higher than those in the LD, HD and SD meadows (**Table 2**; *P*< 0.05) but not significantly different from those in MD meadows (**Table 2**; *P* > 0.05).

**Table 2:** Changes of plant diversity index in different degraded alpine meadows.

Number of experimental sites	Degraded degree	Simpson index	Shannon-Wiener index	Pielou index
1	ND	0.88 ± 0.01a	2.65 ± 0.08a	0.84 ± 0.01ab
2	LD	0.76 ± 0.05b	1.81 ± 0.17b	0.79 ± 0.04b
3	MD	0.90 ± 0.01a	2.47 ± 0.09a	0.87 ± 0.01a
4	HD	0.71 ± 0.01b	1.63 ± 0.03b	0.70 ± 0.02c
5	SD	0.68 ± 0.03b	1.56 ± 0.15b	0.77 ± 0.01bc

Different lowercase letters indicate that the plant diversity index of different degradation degrees was significantly differed at 0.05 level.

The aboveground biomass of the grasses in the MD meadows was highest (19.96 g/m^2^) and significantly higher than that of the grasses in the SD meadows (**Figure 1**; *P*< 0.05). By contrast, the aboveground biomass of sedges was maximum in the LD meadows (54.75 g/m^2^; **Figure 1**). The biomass of forbs in the black-soil-type of degraded meadow was significantly higher than that of forbs in the degraded meadows (**Figure 1**; *P*< 0.05).

### Density and diversity index values of bud banks in the degraded alpine meadows

**Figure 2:**
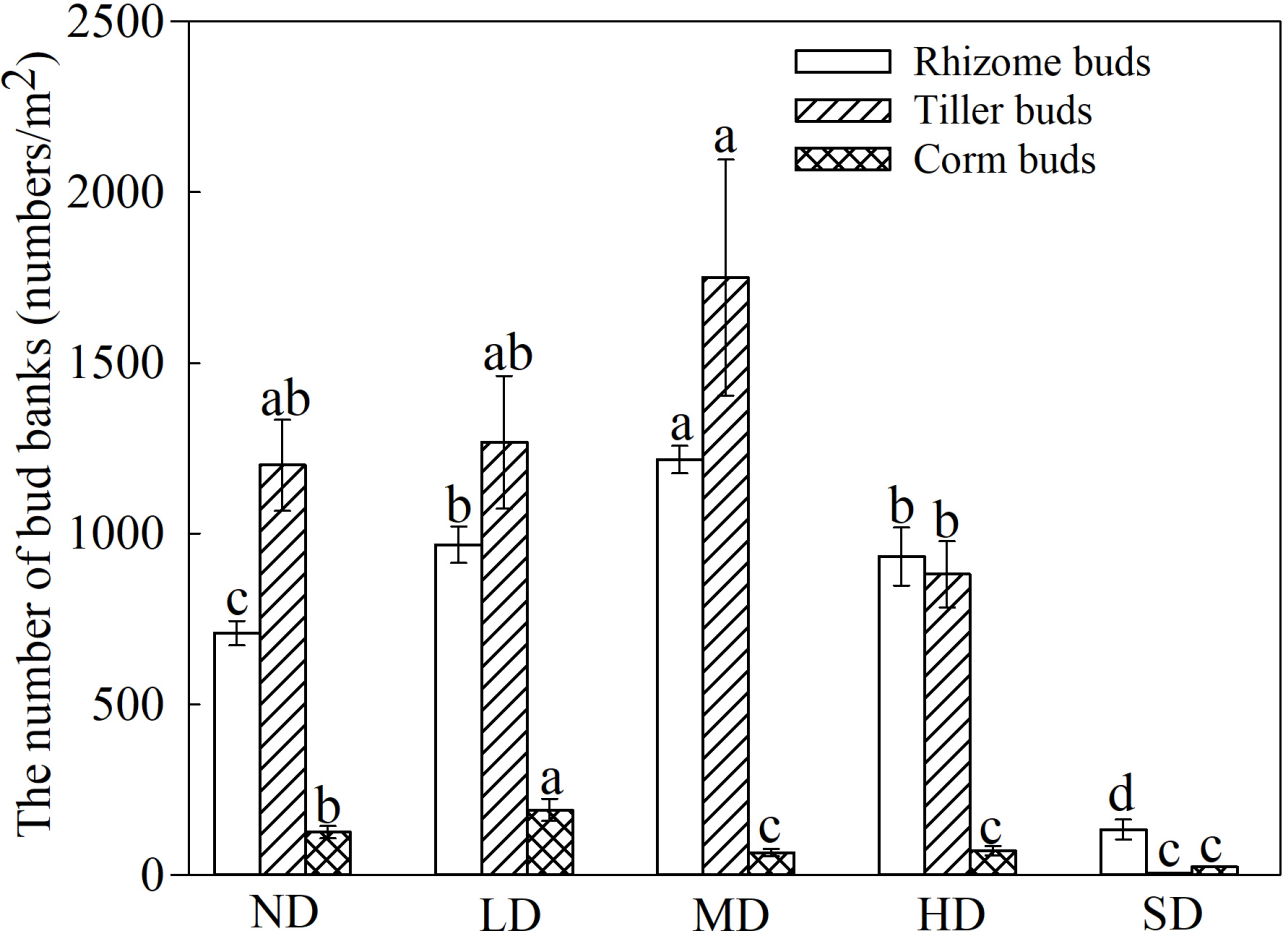
Types and densities of bud banks in degraded alpine meadows. The different lowercase letters indicate that the bud bank density of the same type of alpine meadow significantly differed at the 0.05 level between the different degrees of degradation.

The densities of rhizome, tiller, and corm buds differed among the degraded alpine meadows (**Figure 2**); and the rhizome and tiller bud densities were highest in the MD meadows (average 1216/m^2^ and 1750/m^2^), whereas the rhizome, tiller, and corm bud densities were lowest in the SD alpine meadows (**Figure 2**; *P*< 0.05). Furthermore, the rhizome and tiller buds were higher than the corm buds in the ND, LD, MD and HD alpine meadows (**Figure 2**; *P*< 0.05). The direct examination of bud bank formed plant species in the degraded meadows revealed that 25 species produced buds, 18 and 16 species produced rhizome and tiller buds, respectively, and only 5 species produced corm buds (**Supplement Table S1**). It was noted that most grasses and sedges simultaneously sprouted rhizome and tiller buds; similarly, some forbs such as *Galium verum* and *Potentilla anserina* produced rhizome and corm buds (**Supplemental Table S1**).

Significant changes in bud bank diversity were found among the degraded meadows (**Table 3**; *P*< 0.05). The Margalef and Shannon-Wiener index values of rhizome buds were highest in the ND meadows (1.82 and 1.35, respectively). The Simpson index value of rhizome buds was lowest in the LD meadows (0.13), and the Pielou index value of rhizome buds was highest in the SD meadows (0.91). Similarly, the Margalef, Simpson, Shannon-Wiener, and Pielou index values of tiller buds were highest in the ND meadows (1.49, 0.64, 1.39, and 0.73, respectively). Furthermore, the Margalef, Simpson, Shannon-Wiener, and Pielou index values of corm buds were highest in the ND meadows (0.82, 0.45, 0.73, and 0.8, respectively), while the Shannon-Wiener index value of the corm buds was absent from the HD and SD meadows (**Table 3**).

**Table 3:** Diversity of bud banks of alpine meadow in different degrees of degradation.

Type of bud bank	Diversity index	ND	LD	MD	HD	SD
Rhizome	Margalef	1.82 ± 0.34a	0.53 ± 0.18c	1.11 ± 0.07ab	1.21 ± 0.11ab	0.77 ± 0.27bc
	Simpson	0.60 ± 0.15a	0.13 ± 0.04b	0.66 ± 0.05a	0.56 ± 0.08a	0.52 ± 0.18ab
	Shannon-Wiener	1.35 ± 0.34a	0.30 ± 0.10c	1.30 ± 0.12a	1.09 ± 0.09ab	0.80 ± 0.28bc
	Pielou	0.68 ± 0.13a	0.27 ± 0.09b	0.78 ± 0.05a	0.66 ± 0.08a	0.91 ± 0.31a
Tiller	Margalef	1.49 ± 0.21a	0.26 ± 0.02c	0.74 ± 0.06b	0.26 ± 0.02c	–
	Simpson	0.64 ± 0.14a	0.29 ± 0.01c	0.56 ± 0.05ab	0.32 ± 0.11bc	–
	Shannon-Wiener	1.39 ± 0.29a	0.46 ± 0.01bc	0.98 ± 0.09a	0.49 ± 0.13b	–
	Pielou	0.73 ± 0.14a	0.66 ± 0.02a	0.71 ± 0.07a	0.70 ± 0.18a	–
Corm	Margalef	0.82 ± 0.30a	0.53 ± 0.23a	0.46 ± 0.15a	–	–
	Simpson	0.45 ± 0.17a	0.39 ± 0.13a	0.20 ± 0.07a	–	–
	Shannon-Wiener	0.73 ± 0.28a	0.64 ± 0.22a	0.35 ± 0.12a	–	–
	Pielou	0.80 ± 0.27a	0.74 ± 0.26a	0.50 ± 0.17a	–	–

Different lowercase letters indicate that the diversity index of the same type bud in different degraded alpine meadows is significantly different at 0.05 level. “-” indicates that the diversity index of bud bank is absent.

### Correlations between plant and bud banks among the degraded alpine meadows

**Figure 3:**
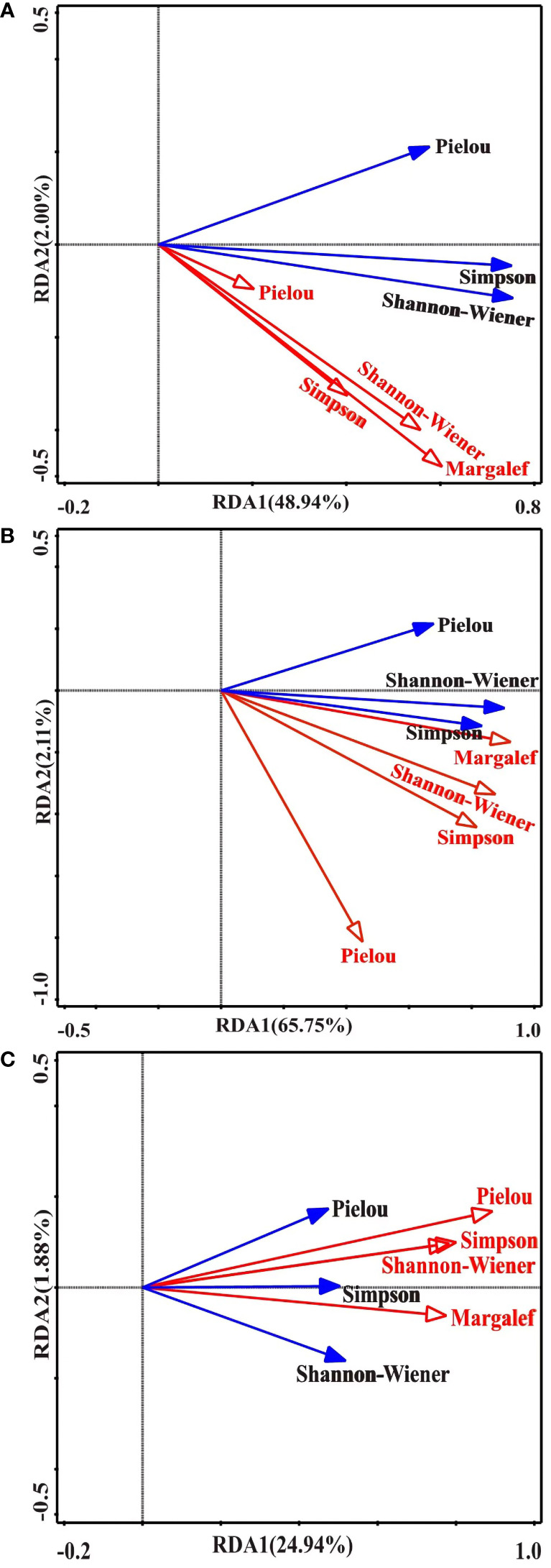
Redundancy analysis (RDA) of plant and bud bank diversity. **(A)** RDA between rhizome buds and plant diversity. **(B)** RDA between tiller buds and plant diversity. **(C)** RDA between corm buds and plant diversity. The blue solid line and black letters indicate the Simpson, Shannon-Wiener, and Pielou index values of the plant community, respectively. The red solid line and red letters indicate the Margalef, Simpson, Shannon-Wiener, and Pielou index values of bud banks, respectively.

The RDA revealed that the Shannon-Wiener, Simpson, and Pielou index values of plants positively correlated with the rhizome, tiller, and corm bud diversities (**Figure 3**, **Supplement Table S2**). The Shannon-Wiener index values of plants correlated positively with the diversity of rhizome buds (**Figure 3A**, **Supplement Table S2**), and the Simpson index values of plants strongly correlated with the diversities of tiller and corm buds (**Figures 3B, C**, **Supplement Table S2**). In addition, the correlation between the diversities of rhizome and tiller buds and the Shannon-Wiener values index of plants were ranked as follows: Margalef > Shannon-Wiener > Simpson > Pielou (**Figures 3A, B**), whereas the correlations between the diversity of corm buds and the Shannon-Wiener index values of plants ranked as follows: Pielou > Simpson > Shannon-Wiener > Margalef (**Figure 3C**).

**Figure 4:**
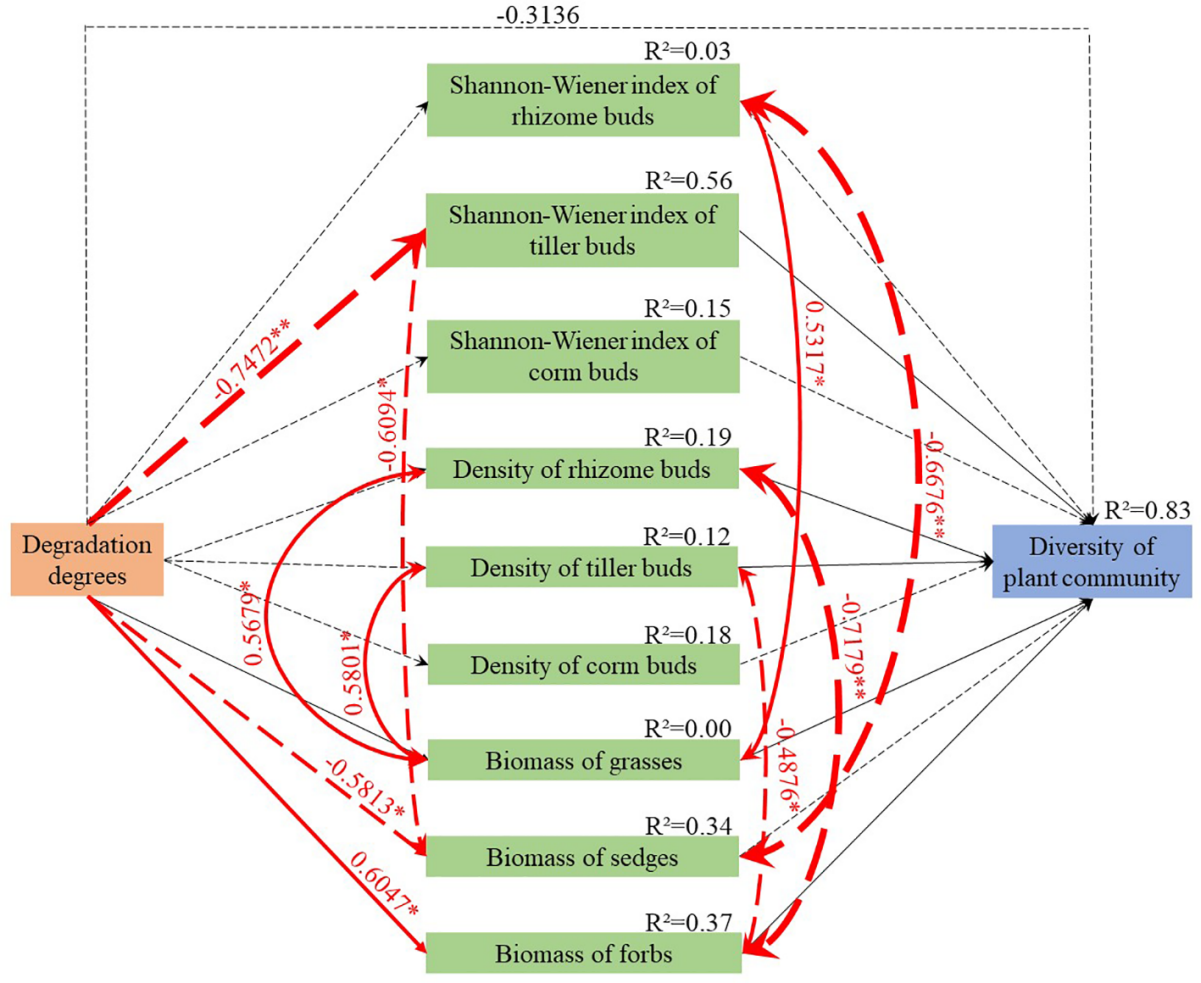
Structural equation model for estimating the correlations between functional group biomass, bud bank density, and alpine meadow diversity in degradation stages. The black dotted line indicates the negative correlation between the variables, and the black solid line indicates the positive correlation between the variables. The red dotted line indicates the significant negative correlation between the variables, and the red solid line indicates the significantly positive correlation between the variables. The values above the lines are the normalized path coefficients of the variables, which indicate the strength of the correlationship (*P< 0.05, **P< 0.01). The width of the line and values above the line are identical to the strength of the correlationship. *R* ^2^ indicates the proportion of the interpreted variance. Goodness of fit statistics: AIC = 78.984, BIC = 100.934, Fisher’s C = 16.984, and *P* = 0.257 > 0.05.

The SEM revealed that the Shannon-Wiener index values of rhizome and corm buds and the density of bud banks decreased as the degradation levels of the meadows intensified (**Figure 4**, **Supplement Table S3**). The Shannon-Wiener index of tiller buds and the aboveground biomass of sedges were also significantly reduced (−0.7472 and −0.5813, respectively). By contrast, the aboveground biomass of the forbs increased as the degree of degradation intensified (0.6047). Furthermore, the aboveground biomass of grasses correlated positively with the densities of rhizome and tiller buds (0.5679 and 0.5801, respectively). However, the aboveground biomass of forbs correlated negatively with the densities of rhizome and tiller buds (−0.7179 and −0.4876, respectively). The decreased biomass of the sedges improved the diversities of rhizome and tiller buds, while the decreased biomass of grasses reduced the diversity of rhizome buds. In conclusion, the density and diversity of bud banks and the biomass of sedges were reduced as the degradation of the meadows intensified, while the decreased diversity of tiller buds, the density of rhizome and tiller buds, and the biomasses of grasses and forbs decreased the diversity of the plant community (**Figure 4**).

## Discussion

Bud banks have been considered the dominant driver of plant regeneration and reproduction, and also play crucial roles in regulating plant composition, community succession and ecosystem function stabilization (Hartnett et al., 2006; Pausas et al., 2018; Li et al., 2023). Different types of sexual reproduction mainly through matured seeds, clonal organs, and branches sprouting buds (i.e. mainly through matured seeds, clonal organs and branch-sprouting buds) have the advantage of absorbing nutrients and water from infertile soil, and nutrients stored in buds also facilitate maternal plants’ resistance or adaptation to drought, cold, trampling, and other biotic or abiotic stresses (Li et al., 2023). In addition, dormant buds provide substantial ecological benefits in maintaining plant community structure due to their low metabolic consumption and maintenance costs, and plant reproduction through new buds is the most effective and low-cost strategy in responding positively to adverse environmental conditions (Ott et al., 2019). Therefore, the reproduction of new individuals through buds and plant population niches maintained depending on bud banks is an important ecological process for species survival and proliferation in alpine meadows (Dalgleish and Hartnett, 2009; Ott et al., 2019). Furthermore, bud banks are also a key force in the rehabilitation of fragmented vegetation and the regulation of positive vegetation succession in alpine meadows (Fidelis et al., 2014). Our findings indicate that both the higher densities of rhizome and tiller buds and the diversity of tiller buds could facilitate the accumulation of greater biomasses of grasses and forbs. Consequently, in this study, greater community biodiversity was maintained in the degraded alpine meadows. To some extent, the moderate disturbances caused by herbivores through feeding and trampling play positive roles in producing more tillers or rhizomes for grasses and sedges. Thus, in this study, the diversity index values of plant and bud banks were markedly higher in the LD or MD meadows than in the other degraded meadows. Therefore, bud banks should be regarded as the original driving force in renewing plant offspring and maintaining community stability; bud banks with higher diversity may facilitate the rehabilitation of degraded meadows.

### Differences in functional group biomass and plant diversity in degraded alpine meadows

Our findings indicate that the biomass of functional groups differs largely among degraded alpine meadows and that the grass biomass in MD meadows is significantly higher than that in other degraded meadows. One possible reason for this is that the new rhizome node in sedges and the density of tillers in grasses would be largely increased after undergoing a moderate grazing disturbance (Li et al., 2015). Meanwhile, herbivores’ preference for feeding on bunch grasses creates better survival and growth conditions for regenerated buds, with less competitivity (Dalgleish and Hartnett, 2006). In the following spring, the niche widths of grasses and sedges were persistently expanded in the MD meadows, whereas that of forbs was reduced (Jiao et al., 2018). By contrast, the biomass of forbs was significantly higher in the SD alpine meadows than in the other degraded meadows. One possible reason for this may be related to the following: the dominant species of native vegetation, such as grasses and sedges, was replaced by toxic weeds in the SD meadows, and the regenerative capacities of rhizome and tiller buds were completely exhausted because of the combined effects of adverse disturbances such as climate warming, overgrazing, and damage by rodents, which facilitated the seed germination of short-lived weeds from the soil seed bank after rainfall in autumn (Li et al., 2011).

Plant diversity differed largely among the degraded alpine meadows; the Shannon-Wiener index values of the ND and MD alpine meadows were significantly higher than those of the LD, HD and SD meadows, while the Simpson and Pielou index values were highest in the MD alpine meadows. These changes may be attributed to the selective feeding behavior of herbivores, which increased the feeding frequency for palatable grasses such as *Elymus nutans* and *Festuca ovina* in the MD meadows. By contrast, the feeding intensities in forbs and sedges largely decreased, providing better regenerative opportunities for forbs and sedges, especially for some poisonous or unpalatable species (Herben et al., 2018). Thus, the Simpson and Pielou index values were higher in the MD meadows than in the other degraded meadows (Wu et al., 2014). Furthermore, the Shannon-Wiener index value was highest in the ND alpine meadows, which may be related to the markedly lower intensity of disturbances by herbivores in the ND meadows than in the degraded meadows. Eliminating the disturbances caused by herbivores also facilitated plant regeneration and maintained higher plant diversity in the ND meadows. Meanwhile, the absence of disturbances by herbivores provided advantages in accelerating regeneration and improving the diversity of bud banks in the ND meadows, as the high intensity of herbivore feeding strongly suppresses the renewal capacity of bud banks. However, bud regenerative ability can be recovered after the adverse effects of herbivore feeding are removed (Ott et al., 2019). In addition, complicated rhizomes and root nets established through renewal buds can absorb abundant nutrients from soils and transfer them to maternal plants, resulting in latter’s growth. The sprouting of new seedlings from buds is thus accelerated, resulting in improving plant diversity (Deng et al., 2014; Herben et al., 2018).

### Differences in the composition of bud banks among the degraded alpine meadows

Both bud type and density varied significantly among the degraded alpine meadows. Bud density was highest in the MD meadows. This finding is consistent with the outcome of maintaining higher plant diversity in the MD meadows, as moderate disturbances from herbivore feeding and trampling are conducive to grass’s greater production of tillers and rhizomes. The renewal tillers or rhizomes expand their niche widths to facilitate resource utilization and reduce competition with maternal plants for nutrients, water, and other resources (Ding et al., 2019). Therefore, more buds and higher plant diversity emerged simultaneously in the MD meadows (Benson et al., 2004; Ding et al., 2019). In addition, grazing-tolerant and low-input species in the MD meadows have adapted to the negative effects of herbivore grazing and trampling, and their strong root systems facilitated their absorption of more nutrients and water, making grasses and sedges the dominant species in the plant community. Grasses and sedges have the potential to sprout newer buds and branches because the nutrients and water reserved in maternal plants ensure bud regeneration (Zhang D. et al., 2019).

By contrast, bud density was lowest in the black-soil-type degraded meadows, which is attributed mainly to the effects of harsh conditions on plant growth and regeneration (Amine Habib et al., 2014; Zhao et al., 2019). For example, with the combined effects of overgrazing, extreme drought, soil erosion and rodent burrowing, palatable grasses and sedges were completely replaced by poisonous weeds; thus, the renewal capacities of tillers and rhizomes were almost lost (Habib et al., 2014). Furthermore, annual weeds with the traits of fast germination and quick reproduction became dominant species in this habitat; thus, the density of corms sprouting from forbs was outstandingly higher than that of corms sprouting from tiller and rhizome buds (Zhao et al., 2019).

Consistent with the results for bud density, the rhizome, tiller, and corm bud diversities also differed significantly among the degraded meadows, and the diversity index value of rhizomes was lowest in the LD meadows, and the Margalef and Shannon-Wiener index values of rhizome buds in the undistributed meadows were markedly higher than those in the degraded meadows. No significant difference in corm bud diversity was found among the undistributed, LD or MD meadows. A possible reason for this change is that the buffer zone formed by dense litters not only alleviated soil erosion from rainfall but also reduced the evaporation of water from the soil (Sheldrake et al., 2017). This physical safeguard established by litters plays an important role in bud regeneration because water and nutrients are essential factors in the renewal and growth of buds (Guo et al., 2019). Litters also facilitated the colonization of soil microorganisms, as the latter’s main source of carbon is decomposed litters (Liu et al., 2021). In return, numerous soil microorganisms also quickly decompose litters into soil organic matter; consequently, soil with higher nutrients has advantages in the renewal and expansion of buds (Zhu et al., 2016). Furthermore, the combined effects of tiller buds that sprout from the bases of grasses and rhizome buds emerging from sedge nodes can enhance the competitiveness of grasses and sedges and facilitate their development into dominant species in undistributed meadows. These findings are partly explained by the fact that the cover and biomass of sedges and grasses were highest in the undisturbed meadows (Liu et al., 2022). Therefore, the coupled effects of the strong competition of maternal plants and the persistent renewing capacity of buds could be regarded as a virtuous cycle in maintaining a higher diversity of buds and dominating species (Li et al., 2022).

The Simpson index was highest in the MD meadows, possibly because the moderate disturbances caused by herbivores had a positive effect on enhancing the proportion of grasses in the alpine meadow community, and because grasses have the capacity to sprout tiller and rhizome buds. Therefore, grasses demonstrated strong interspecific competitiveness for sunlight, water and nutrients, which contributed to their regenerating more tiller or rhizome buds; the Simpson index values of tiller and rhizome buds increased in the MD meadows (Benson and Hartnett, 2006).

Our study confirms that plant diversity is higher in LD meadows than in HD and black-soil-type degraded meadows, but bud bank diversity is lower in the former than in the latter. This may be due to the growth and development of plants not only depending on the bud bank but also on the soil seed bank for sexual reproduction in the LD meadows (Chen et al., 2022). However, it is difficult to develop seeds into plants because of the increased soil bulk density and decreased water and nutrients content due to the feeding and trampling of livestock and human activities in SD meadows (Zhang W. J. et al., 2019). Furthermore, the wide stems and leaves of forbs prevent seed implantation (Klimešová and Klimeš, 2007; Dong et al., 2015). Therefore, bud banks have become the most effective method for population renewal and expansion.

### Correlation between plant species and bud banks in degraded alpine meadows

Previous studies have indicated that bud bank diversity plays a crucial role in improving plant diversity and enhancing the stability and function of vegetation communities (Ott et al., 2019; Yang et al., 2022). In our study, the Shannon-Wiener index values of rhizome and tiller buds correlated positively with the plant diversity index, and plant diversity and the diversity index values of rhizome and tiller buds were markedly higher in the MD meadows than in the other degraded meadows. It is interesting that the dynamic change in the density of bud banks preceded the changes in plant and bud bank diversities. The possible reasons for these changes may be summarized as follows: (1) The type and density of bud bank plants largely determined the restoration speed of perennial plants after disturbance by abiotic and biotic stresses such as drought, fire, rodent burrowing, and herbivore foraging (Hämälä et al., 2017). Benson and Hartnett (2006) found that more than 99% of aboveground stems are formed by bud banks in grassland after fire, whereas only 1% of plant regeneration is restored through seed banks. Meanwhile, Zhang et al. (2009) suggested that the propagation and regeneration of plant populations through bud banks demonstrates superiority in growth and adaptivity to adverse survival conditions. Therefore, the succession direction of vegetation was largely regulated by the density and type of bud bank. (2) Perennial sedges and grasses are the dominant species in the alpine meadows’ plant community of alpine meadows. Aboveground tissues, including the stems, branches, and leaves of sedges and grasses, withered after their reproduction stage. To ensure the regeneration of tiller or rhizome buds in the next spring, abundant nutrients should be prioritized in the allocation to nutrient storage organs such as roots or buds (Dalgleish and Hartnett, 2009; Carter et al., 2012; Ott and Hartnett, 2014; Zhang D. et al., 2019; Wu et al., 2020b). Dormant buds sprout quickly from the base or rhizome by consuming nutrients stored in buds; consequently, the maternal plant cover increases quickly through the persistent regeneration cycles of tiller and rhizome buds (Wang et al., 2018; Ding et al., 2019). Therefore, bud regeneration was vital to determine vegetation structure and diversity.

Likewise, in our study, the combined effects of greater tiller bud diversity, more rhizome and tiller buds, and higher aboveground biomass of grasses and forbs resulted in higher levels of plant diversity in MD meadows. The probable reasons are as follows: (1) The regeneration rate of the density of tiller buds preceded the changes in bud bank diversity in the degraded alpine meadows, while tiller bud diversity was synchronized with plant diversity. Therefore, the density of tiller buds sprouting from grasses largely determined the diversity of bud banks and grasses in the plant community. It can be concluded that the tiller bud density of grasses could be a predictor of the dynamic change in the plant community diversity (Yu et al., 2020). (2) Grass and sedge coverage in the plant community decreased sharply when grazing intensity exceeded the MD meadows’ capacity; by contrast, toxic weeds demonstrated more interspecific competition and became dominant species in the heavily degraded (HD) and SD meadows (Ott and Hartnett, 2012). Although bud bank diversity was relatively high in the HD meadows, bud banks’ regenerative capacity was decreased; thus, both the diversity and density of bud banks were sharply reduced in the SD meadows, ultimately leading to the complete loss of the vegetation community’s function and regeneration (Ott et al., 2019; Zhao et al., 2019).

## Conclusion

The renewal capacity of perennial herbaceous plants was synchronized with the regenerating and expanded abilities of their bud banks, which is a vital survival strategy for species to adapt to the adverse environments of alpine meadows (Ott and Hartnett, 2015). Compared with the function of buds in alpine species, rhizome buds demonstrated stronger spatial expansion underground and better efficiency in absorbing nutrients from the soil, and tiller buds had an advantage in maintaining native populations; whereas corm buds contributed less to plant renewal (Carter et al., 2012). Our study results suggest that the Shannon-Wiener index values of plants and buds were significantly higher in the undistributed and MD alpine meadows than in the LD, HD, and SD alpine meadows. Similarly, the total bud bank density was highest in the moderately disturbed habitats. In addition, the rhizome, tiller, and corm bud diversities correlated positively with plant diversity. As far as we know, previous studies have focused mainly on evaluating the dynamic succession processes of degraded grasslands by monitoring changes in plant composition and evaluating the diversity levels of vegetation communities (Zhu et al., 2016; Wang et al., 2017; Ott et al., 2019; Yang et al., 2022). Our results suggest that bud banks can be considered, in part, the original driving force for vegetation renewal in alpine meadows and that the renewing capacity of bud banks largely determines degraded meadows’ restorative efficiency and suppresses their degradation. In addition, our findings also provide convincing theoretical support for the idea that the combined effects of accelerating bud regeneration and reseeding grasses are the most efficient methods for restoring quickly degraded meadows (Ott and Hartnett, 2014). Therefore, the substantial role of bud banks, especially for sedges and grasses, should be considered in the restoration of degraded meadows in alpine areas. Moreover, the results of changes in bud bank type and density caused by herbivore disturbance should be interpreted with caution, because other abiotic factors, such as temperature and precipitation are not to be overlooked in determining the bud banks’ traits, and some control experiments regarding the role of other biotic and abiotic factors in regulating bud banks of alpine meadows should be taken into account in future researches.

## Data availability statement

The original contributions presented in the study are included in the article/**Supplementary Material**. Further inquiries can be directed to the corresponding author.

## Author contributions

YL: Writing – original draft. GB: Funding acquisition, Writing – review & editing. PZ: Investigation, Formal Analysis, Writing – review & editing. XF: Data curation, Investigation, Writing – review & editing. JM: Investigation, Methodology, Writing – review & editing. HL: Investigation, Data curation, Writing – review & editing. HS: Methodology, Investigation, Writing – review & editing. XW: Investigation, Methodology, Writing – review & editing. BT: Investigation, Writing – review & editing. KL: Supervision, Writing – review & editing.
