# Supplementary material for: Changes in bud bank and their correlation with plant community composition in degraded alpine meadows
Source: Front Plant Sci. 2023 Oct 13;14:1259340. doi: 10.3389/fpls.2023.1259340 (PMC10613031; doi:10.3389/fpls.2023.1259340)
Supplement: Supplementary file 1 [file Table_1.docx]

Table S1 Types and quantity of bud banks in different degraded alpine meadows

| The density of bud banks | The density of rhizome banks（numbers/m^2^） | | | | | The density of tiller banks（numbers/m^2^） | | | | | | The density of corm banks（numbers/m^2^） | | | | | |  |
| --- | --- | --- | --- | --- | --- | --- | --- | --- | --- | --- | --- | --- | --- | --- | --- | --- | --- | --- |
| Degree of degradation  The types of plants | ND | LD | MD | HD | SD | | ND | LD | MD | HD | SD | | ND | LD | MD | HD | SD | |
|  |  |  |  |  |  | |  |  |  |  |  | |  |  |  |  |  | |
| *Polygonum viviparum* | - | - | - | - | - | | - | - | - | - | - | | 11 | 1 | - | - | - | |
| *Carex capillifolia* | 30 | - | 3 | - | - | | 79 | - | - | 2 | - | | - | - | - | - | - | |
| *Euphorbia esula* | 4 | - | - | - | - | | 1 | - | - | - | - | | - | - | - | - | - | |
| *Carex alatauensis* | 5 | - | 53 | - | - | | 17 | - | 94 | - | - | | - | - | - | - | - | |
| *Elymus nutans* | 1 | 1 | 22 | 42 | - | | 14 | 27 | 95 | 118 | - | | - | - | - | - | - | |
| *Galium verum* | 2 | - | - | - | - | | - | - | - | - | - | | 1 | - | - | - | - | |
| *Ajania tenuifolia* | 2 | - | - | 17 | - | | - | - | - | - | - | | - | - | - | - | - | |
| *Lancea tibetica* | 3 | - | 22 | 2 | - | | - | - | - | - | - | | - | - | - | - | - | |
| *Thermopsis lanceolata* | 25 | - | - | - | - | | 10 | - | - | - | - | | - | - | - | - | - | |
| *Carex atrofusca* | - | 2 | - | - | - | | 4 | - | - | - | - | | - | - | - | - | - | |
| *Potentilla fruticosa* | - | - | - | - | - | | 4 | - | - | - | - | | - | - | - | - | - | |
| *Deschampsia cespitosa* | 5 | - | - | - | - | | 6 | - | - | - | - | | - | - | - | - | - | |
| *Stipa aliena* | - | - | - | - | - | | 1 | - | - | - | - | | - | - | - | - | - | |
| *Carex aridula* | 6 | - | 29 | - | - | | 7 | - | 16 | - | - | | - | - | - | - | - | |
| *Poa annua* | 2 | - | 11 | 12 | - | | - | - | 1 | 64 | - | | - | - | - | - | - | |
| *Potentilla anserina* | - | - | 5 | 6 | 10 | | - | - | - | - | - | | 3 | 12 | 12 | 14 | 5 | |
| *Allium sikkimense* | - | - | - | - | - | | 1 | - | - | - | - | | - | - | - | - | - | |
| *Carex ligulata* | - | 113 | - | - | - | | - | 125 | - | - | - | | - | 35 | - | - | - | |
| *Ptilagrostis dichotoma* | - | - | - | - | - | | - | - | 3 | - | - | | - | - | - | - | - | |
| *Oxytropis ochrocephala* | - | - | 1 | - | - | | - | - | - | - | - | | - | - | - | - | - | |
| *Geranium wilfordii* | - | - | - | - | - | | - | - | 1 | - | - | | - | - | - | - | - | |
| *Gentiana macrophμlla* | - | - | - | - | - | | - | - | - | - | - | | - | - | 1 | - | - | |
| *Spergularia salina* | - | - | - | 30 | - | | - | - | - | - | - | | - | - | - | - | - | |
| *Carex* spp. | - | - | - | 2 | 5 | | - | - | - | - | 1 | | - | - | - | - | - | |
| *Polygonum sibiricum* | - | - | - | 1 | 1 | | - | - | - | - | - | | - | - | - | - | - | |
| Sum | 85 | 116 | 146 | 112 | 16 | | 144 | 152 | 21 | 184 | 1 | | 15 | 48 | 13 | 14 | 5 | |

Note: “-” indicates that the bud is absent.
