# Supplementary material for: Changes in bud bank and their correlation with plant community composition in degraded alpine meadows
Source: Front Plant Sci. 2023 Oct 13;14:1259340. doi: 10.3389/fpls.2023.1259340 (PMC10613031; doi:10.3389/fpls.2023.1259340)
Supplement: Supplementary file 2 [file Table_2.docx]

Table S2 The fit indices (R^2^) for RDA models between the plant diversity indexes (including the Simpson, Shannon-Wiener, and Pielou index values of plant community) and bud banks diversity index (including Margalef, Simpson, Shannon-Wiener, and Pielou index values of rhizome, tiller and corm buds, respectively)

|  | Bud bank diversity | | |
| --- | --- | --- | --- |
|  | Rhizome buds | Tiller buds | Corm buds |
| Plant diversity | 0.85 | 0.78 | 0.84 |
