# Supplementary material for: Changes in bud bank and their correlation with plant community composition in degraded alpine meadows
Source: Front Plant Sci. 2023 Oct 13;14:1259340. doi: 10.3389/fpls.2023.1259340 (PMC10613031; doi:10.3389/fpls.2023.1259340)
Supplement: Supplementary file 3 [file Table_3.docx]

Table S3 The fit indices for SEM models among functional group biomass, bud bank density and alpine meadow diversity in different degradation stages

| Fit indices | AIC | BIC | Fisher's C | *P*-value |
| --- | --- | --- | --- | --- |
| Value | 78.98 | 100.93 | 16.98 | 0.26 |

Note: AIC is the abbreviation of Akaike Information Criterion, which is a mathematical method for evaluating how well a model fits the data it was generated from, lower value of AIC indicated the model better fits the data. BIC is the abbreviation of Bayesian information criterion, which is a criterion for [model selection](https://en.wikipedia.org/wiki/Model_selection) among a finite set of models; models with lower BIC are generally preferred. Fisher's C is used to test whether the results of a randomized experiment support a hypothesis about a randomized experiment. Smaller value of Fisher's C indicated that selected model is better others.
